# Supplementary material for: Omental Macrophagic “Crown-like Structures” Are Associated with Poor Prognosis in Advanced-Stage Serous Ovarian Cancer
Source: Curr Oncol. 2021 Oct 19;28(5):4234–46. doi: 10.3390/curroncol28050359 (PMC8534828; doi:10.3390/curroncol28050359)
Supplement: Supplementary file 1 [file curroncol-28-00359-s001.zip › curroncol-1362866-supplementary.pdf]

Current Oncology

MDPI

Article

Omental Macrophagic "Crown-Like Structures" Are Associated with Poor Prognosis in Advanced-Stage Serous Ovarian Cancer

Yu-Ling Liang [1,†], Chang-Ni Lin [1,†], Hsing-Fen Tsai [1], Pei-Ying Wu [1], Sheng-Hsiang Lin [2], Tse-Ming Hong [2,*] and Keng-Fu Hsu [1,2,*]

Supplementary Materials

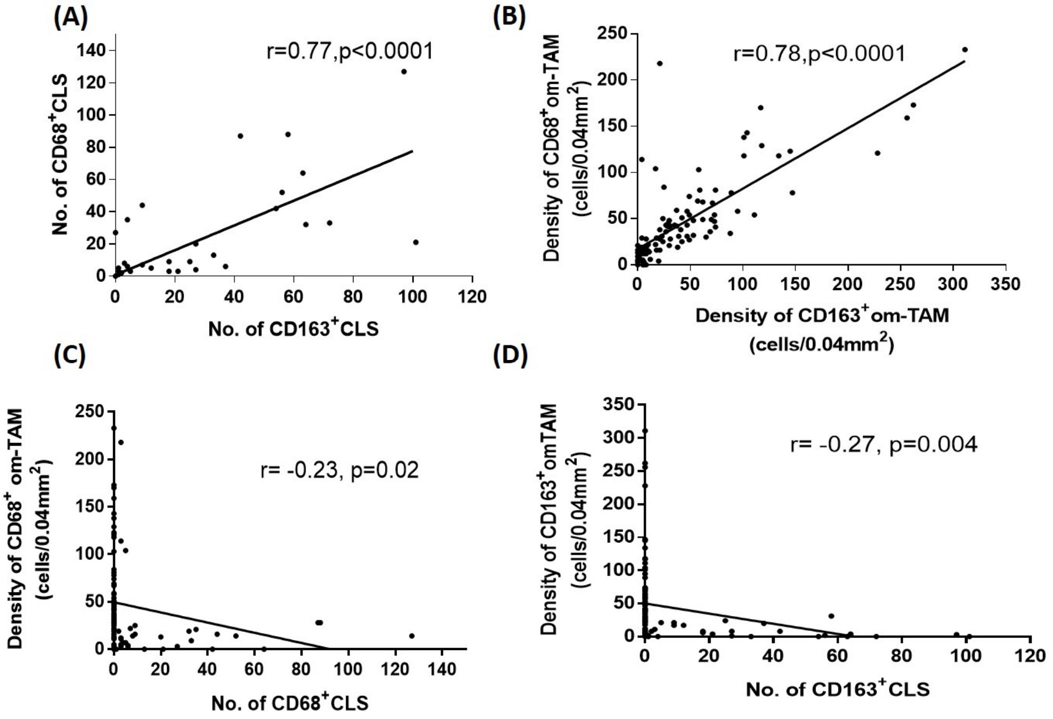

Figure S1. The number of omental CD68+ CLSs exhibited a positive correlation with omental CD163+CLSs; the density of CD68+ om-TAMs had a positive correlation with the density of CD163+om-TAMs. There was little correlation between the density of CD68+ om-TAMs and CD68+ CLSs or between the density of CD163+om-TAMs and the number of CD163+ CLSs. (A) There was a strong positive correlation between the number of omental CD68+ CLSs and the number of omental CD163+ CLSs (r = 0.77, p<0.0001). (B) There was a strongly positive correlation between the density of CD68+ om-TAMs and CD163+ om-TAMs (r = 0.78, p<0.0001). (C) There was little correlation between the density of CD68+ om-TAMs and CD68+ CLSs (r = -0.23, p=002). (D) There was little correlation between the density of CD163+ om-TAMs and the number of CD163+ CLSs (r =-0.271, p= 0.004).

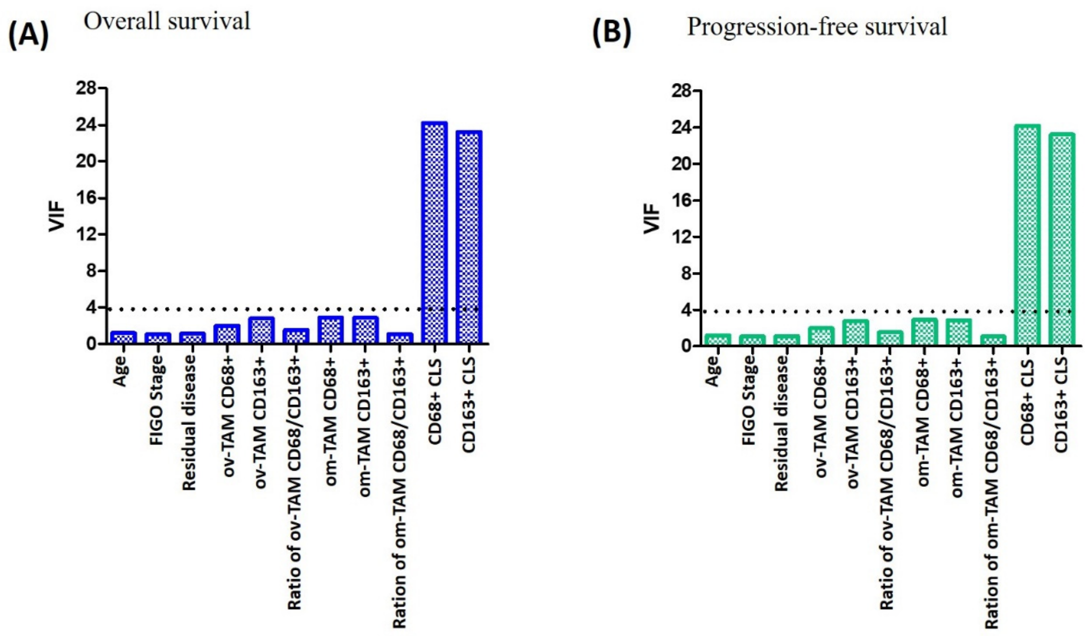

Figure S2. There was a high degree of multicollinearity for $CD68^+$ CLSs and $CD163^+$ CLSs in the model for OS and PFS. (A) The VIFs of $CD68^+$ CLSs and $CD163^+$ CLSs in OS were 24.22 and 23.22, respectively. (B) The VIFs of $CD68^+$ CLSs and $CD163^+$ CLSs in PFS were 24.1 and 23.2, respectively.

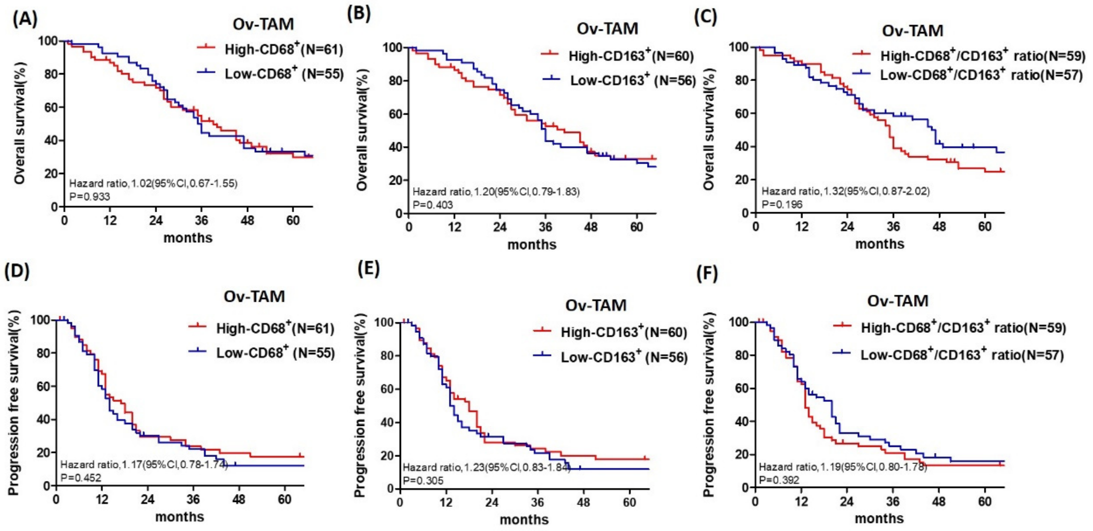

Figure S3. In advanced-stage HGSOC, the ovarian tumor density of $CD68^+$ or $CD163^+$ ov-TAMs was not associated with patient prognosis. (A, B) For OS analysis, neither the density of $CD68^+$ nor $CD163^+$ ov-TAMs was associated with patient OS (p=0.933; p=0.403, respectively). (D, E) For PFS analysis, neither the density of $CD68^+$or $CD163^+$ ov-TAMs was associated with patient PFS (p=0.452; p=0.305, respectively). (C, F) The ratio of primary ovarian tumor $CD68^+$/ $CD163^+$ TAM was not significantly associated with OS or PFS (p=0.196; p=0.392).

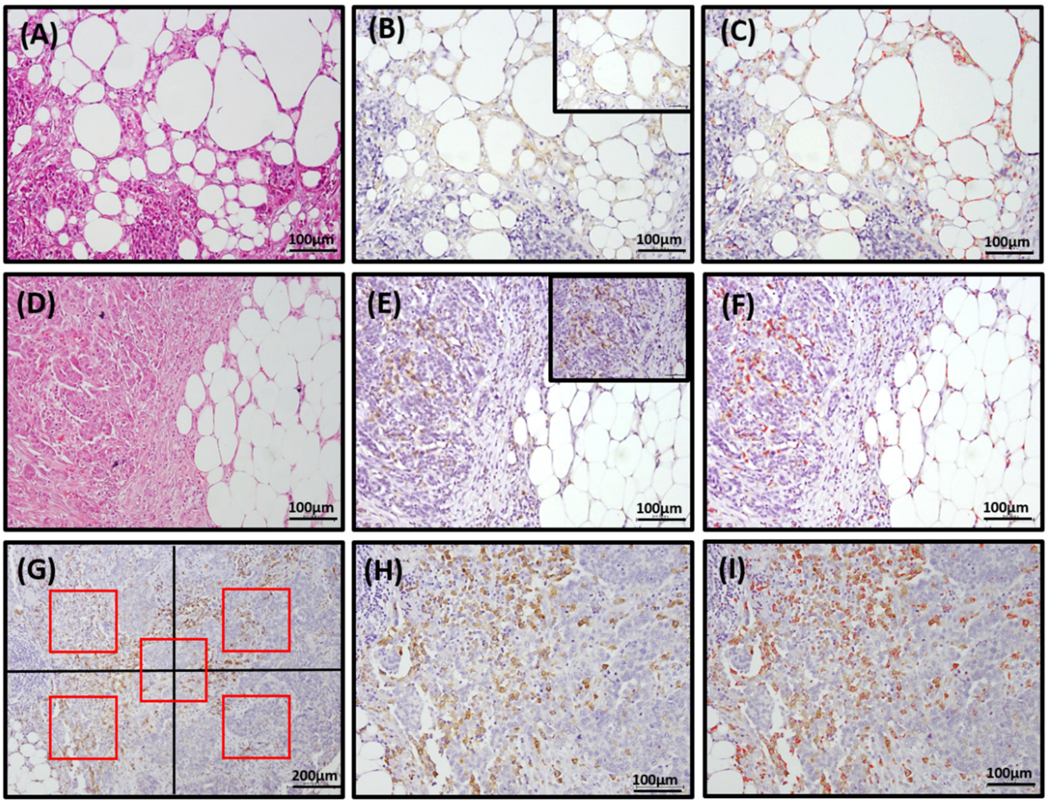

Figure S4. Representative image of CLS and TAMs by immunohistochemical staining for CD163 and ImageJ software-assisted images. A, D, and G, H&E; B, E, and H, IHC CD163 staining; C, F and I, ImageJ images. In (C), note that there are five adipocytes completely surrounded by CD163-positive macrophages, counted as five $CD163^{+}$ CLSs. (G) For TAM density, the area with the greatest concentration of CD163-stained TAMs was identified. Then, five fields (red box, four quadrants and a central area, each field contained 0.04 mm^2) under 100 x magnification were selected, and the number of $CD163^{+}$ macrophages was counted under 200 x magnification with the assistance of ImageJ software (I).

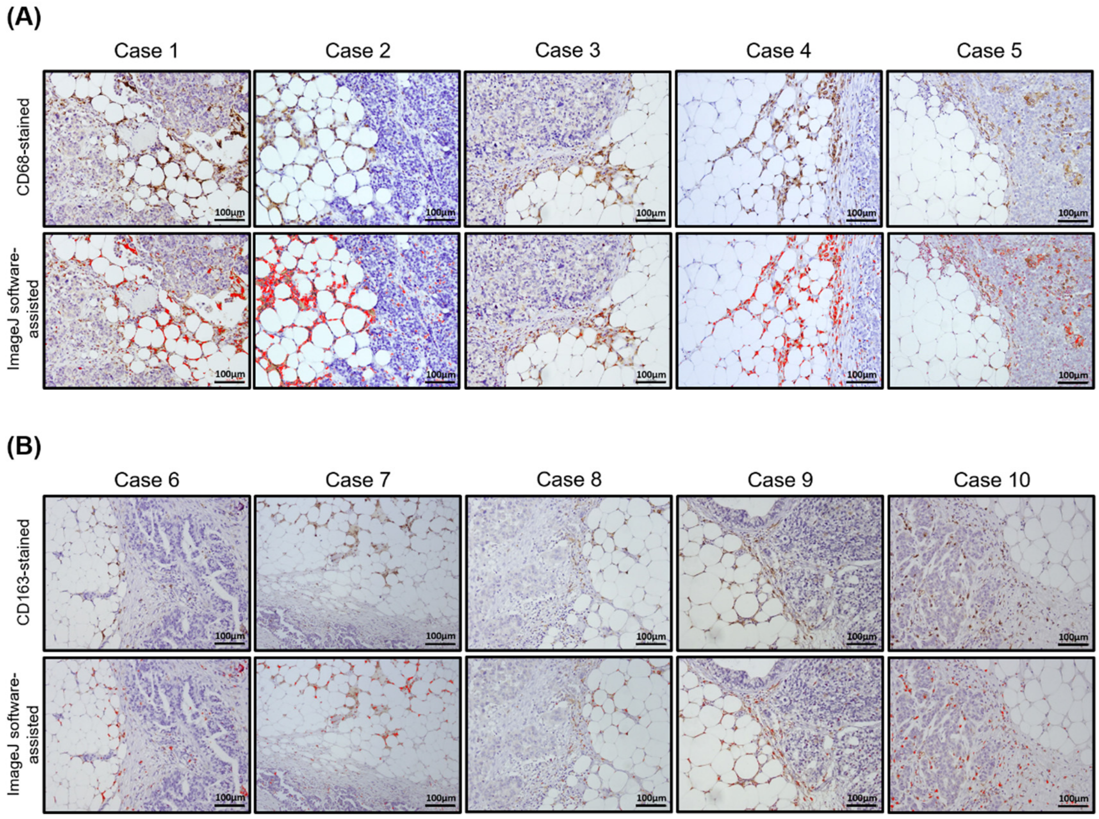

Figure S5. Ten cases of different counts of CLSs existence in omentum after CD68 (A, upper) or CD163 (B, upper) staining. Lower panels of pictures were CD68- or CD163-positive macrophages quantitatively by ImageJ software to perform CLS number calculation.

Table S1. Association between clinical parameters and primary ovarian tumor TAM in 116 advanced stage serous ovarian cancer patients.

Variable	Total N=116	CD68⁺ ov-TAM High (N=61)	CD68⁺ ov-TAM Low (N=55)	CD68⁺ ov-TAM P value	CD163⁺ ov-TAM High (N=60)	CD163⁺ ov-TAM Low (N=56)	CD163⁺ ov-TAM P value	Ratio of CD68⁺/CD163⁺ ov-TAM High (N=59)	Ratio of CD68⁺/CD163⁺ ov-TAM Low (N=57)	Ratio of CD68⁺/CD163⁺ ov-TAM P value
**Age(y/o)**										
Mean (range)	57.2 (23~88)	55.3 (23~88)	58.9 (44~82)	0.16	55.4 (23~88)	58.8 (36~82)	0.38	58.8 (30~88)	55.2 (23~84)	0.61
**BMI**										
Mean (range)	23 (15~34)	23 (18~34)	22 (15~34)	0.08	24 (18~34)	22 (15~29)	0.03	23 (15~34)	23 (16~34)	0.51
**FIGO stage**				1			1			0.78
IIIA	4 (3)	3 (5)	1 (2)		2 (3)	2 (4)		2 (3)	2 (3)	
IIIB	17 (15)	6 (10)	11 (20)		9 (15)	8 (14)		11 (19)	6 (11)	
IIIC	82 (71)	45 (74)	37 (67)		42 (70)	40 (71)		40 (68)	42 (74)	
IVA	9 (8)	5 (8)	4 (7)		4 (7)	5 (9)		5 (8)	4 (7)	
IVB	4 (3)	2 (3)	2 (4)		3 (5)	1 (2)		1 (2)	3 (5)	
**Residual disease**				1			0.57			0.57
optimal(≤1 cm)	71 (61)	37 (61)	34 (62)		34 (57)	37 (66)		38 (64)	33 (58)	
Not-optimal(>1 cm)	45 (39)	24 (39)	21 (38)		26 (43)	19 (34)		21 (36)	24 (42)	
**Chemotherapy**				0.70			0.85			0.85
sensitive	70 (60)	38 (63)	32 (58)		37 (62)	33 (60)		35 (59)	35 (61)	
resistant	42 (36)	21 (34)	21 (38)		21 (35)	21 (38)		22 (38)	20 (35)	
No chemotherapy	4 (4)	2 (3)	2 (4)		2 (3)	2 (2)		2 (3)	2 (4)	
